# Chimeric Antigen Receptor (CAR) T Cell Therapy for Neuromuscular Disorders: A Systematic Review

**DOI:** 10.7759/cureus.93017

**Published:** 2025-09-23

**Authors:** Josef Finsterer

**Affiliations:** 1 Neurology, Neurology and Neurophysiology Center, Vienna, AUT

**Keywords:** car t cell therapy, immunological, myasthenia, neuromuscular disorders, t-cells

## Abstract

Case reports and case studies increasingly demonstrate that chimeric antigen receptor (CAR) T cell therapy (CTCT) is beneficial not only in hematologic malignancies but also in immunologic diseases, including neuromuscular disorders. The aim of this review is to provide an overview of the current status of CTCT in immune-mediated neuromuscular disorders. This is a systematic review of relevant literature recruited using PubMed, Embase, Scopus, and Google Scholar search terms.

Neuromuscular disorders for which CTCT has been used to date include myasthenia gravis (n = 4), Lambert-Eaton syndrome (n = 1), myasthenia/Lambert-Eaton overlap (n = 2), dermatomyositis (n = 2), immune-mediated necrotizing myositis (n = 2), idiopathic inflammatory myopathy (n = 1), anti-synthetase syndrome (n = 4), and chronic inflammatory demyelinating polyneuropathy (n = 2). In most cases, CTCT was directed against CD19-positive lymphocytes and in some cases against B cell maturation antigen. In all reported patients, there was a significant improvement in motor function and quality of life, with some even making a full recovery several months after the application of CTCT.

In conclusion, CTCT appears to be a promising therapeutic option for patients with severe immune neuromuscular disorders in whom previous treatment with multiple immunomodulatory therapies has been ineffective. CTCT should be considered in patients with immune neuromyopathy who do not respond to immunomodulatory therapies.

## Introduction and background

Chimeric antigen receptor (CAR) T cell therapy (CTCT) was originally developed for the treatment of carcinomas and hematologic malignancies [1] but is now also used for various immunological diseases, including diseases of the central nervous system (CNS) and peripheral nervous system (PNS) [2]. In preclinical and clinical studies, there is increasing evidence that various PNS diseases can benefit from CTCT even when immunomodulatory therapies have previously failed [3]. CTCT as an initial treatment for neuromuscular disorders (NMDs) prior to the use of immunomodulatory therapies has not yet been tested. CTCT has been used in a small number of patients with myasthenia gravis (MG) [4-7], myasthenic syndrome, also known as Lambert-Eaton syndrome (LES) [6,8], idiopathic immune myositis (IIM) such as dermatomyositis (DM) [9,10], immune-mediated necrotizing myopathy (IMNM) [11], and anti-synthetase syndrome (ASS) [12-14] as well as chronic inflammatory demyelinating polyneuropathy (CIDP) [15].

CTCT was originally developed as a cancer immunotherapy in which the patient's own T cells are used to fight and destroy cancer cells [16]. For this purpose, these T cells are genetically modified in the laboratory so that they express the CAR, which enables them to recognize and bind to specific proteins (antigens) on cancer cells or B lymphocytes. After modification, these CAR T cells are multiplied and infused into the patient, where they can attack and kill the cancer cells or antibody-producing immune cells [17]. Prior to infusion, preconditioning with fludarabine or cyclophosphamide is performed to deplete the B cells [17]. In immunological diseases, CAR T cells destroy the B cells and prevent them from producing autoantibodies that can attack various structures. The rationale behind this approach is that profound depletion of B cells, including autoreactive B cell clones, could restore normal immune function, which is referred to as immune reset [18]. In order to collect, modify, and redistribute the T cells, patients scheduled for CTCT must remain hospitalized for four to six weeks. Side effects of CTCT include allergic reactions; cytokine release syndrome (CRS); immune effector cell-associated neurotoxicity syndrome (ICANS) characterized by confusion, disorientation, and epilepsy; increased risk of infection; and tumor lysis syndrome. This systematic review aims to provide an overview of the current status of CTCT in immune-mediated NMDs.

## Review

Methods

**Figure 1:**
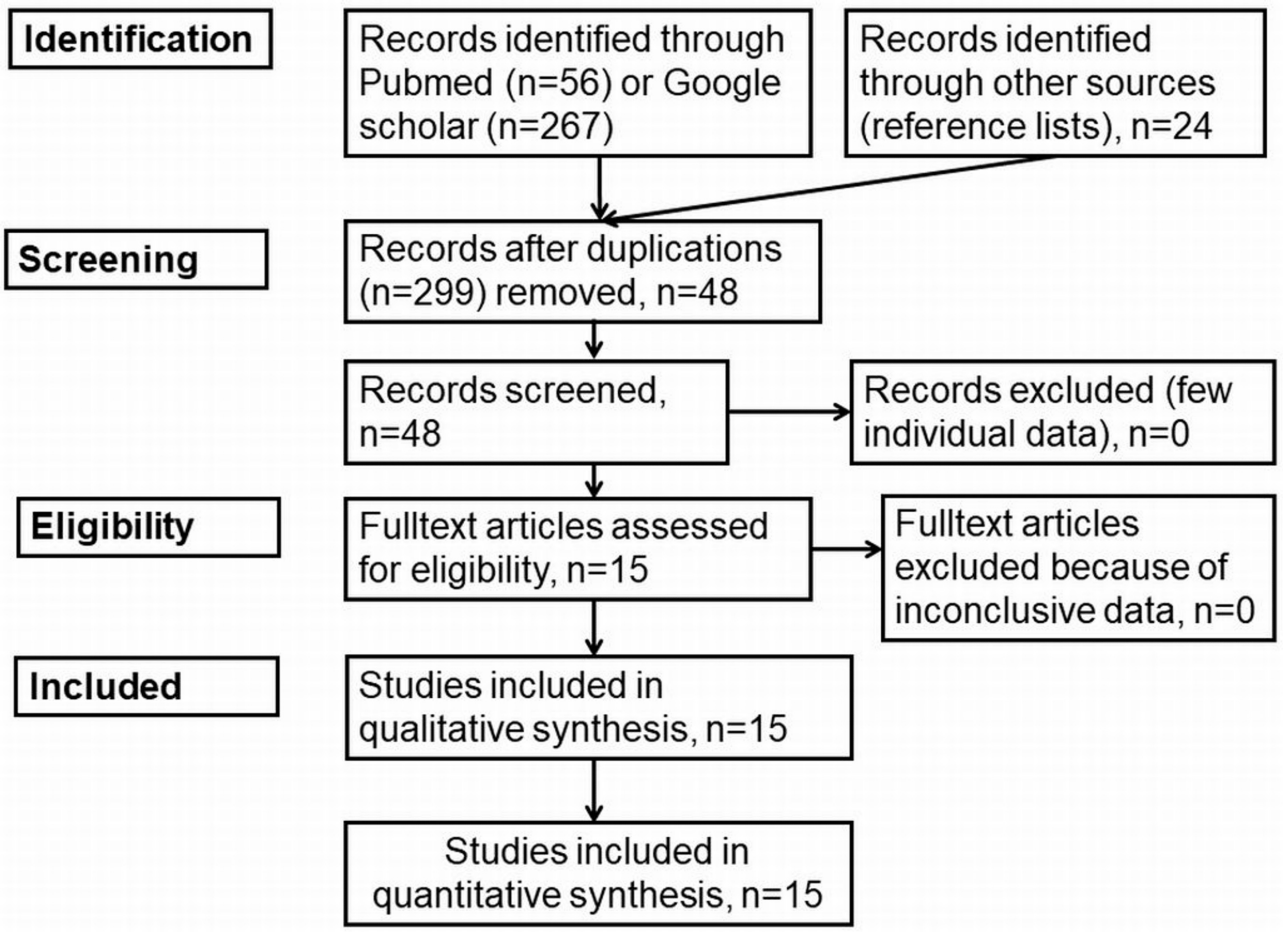
Identification of studies via databases and registries

A literature search was conducted in the PubMed and Google Scholar databases by a single reviewer to find and select relevant literature using the search terms “CAR-T cell therapy” in combination with “neuromuscular diseases”, “myasthenia gravis”, “Lambert-Eaton myasthenic syndrome”, “idiopathic immune myositis”, “chronic inflammatory demyelinating polyneuropathy”, and “dermatomyositis”. No restrictions were made in terms of gender, ethnicity, language, nation, or context. Articles were excluded if they were not available as a complete article, did not meet the search criteria, or did not contain original data but only provided an overview of the topic. The search was carried out between June and September 2025. A total of 56 articles were initially identified via PubMed and 267 via Google Scholar (Figure 1). In addition, Scopus and Embase databases were screened for articles meeting the inclusion criteria. Two hundred ninety-nine articles were excluded due to duplication, all were available as full text, and 15 were reviews or abstracts (Figure 1). Finally, 15 papers were included in the analysis [3-8,10,11,13-15,19-24]. No statistical analysis was performed. The review was not registered in PROSPERO.

Results

Myasthenia Gravis

The first MG patient treated with CTCT was a 64-year-old man reported in 2022 by Zhang et al. [4]. The patient had been diagnosed with MG eight years earlier and treated with pyridostigmine, to which prednisone was added a few months later due to ineffectiveness. During a myasthenic crisis, the patient required intravenous immunoglobulins (IVIG) and was placed on tacrolimus [4]. Despite this treatment, myasthenic crises recurred, and a tracheostomy had to be performed [4]. The MG was classified as refractory, and CTCT was initiated. Although the patient experienced some transient side effects, he improved steadily until the seventh month of follow-up (Tables 1, 2) [4].

**Table 1:** Neuromuscular disorders having been treated with CTCT ASS: anti-synthetase syndrome; CR: complete remission; CTCT: CAR T cell therapy; NMD: neuromuscular disorder; nr: not reported; PR: partial remission; MG: myasthenia gravis; LES: Lambert-Eaton syndrome; DM: dermatomyositis; IMNM: immune-mediated necrotizing myopathy; IIM: idiopathic immune myositis; CIDP: chronic inflammatory demyelinating polyneuropathy; CK: creatine kinase; CRP: C-reactive protein

NMD	Antigen	Age/sex	Target	Outcome	Reference
MG	AchR	64/m	CD19, BCMA	CR by 4 months	Zhang et al., 2024 [4]
MG	AchR	33/f	nr	CR by 12 months	Tian et al., 2024 [5]
MG	MUSK	60/f	nr	PR by 3 months	Tian et al., 2024 [5]
MG/LES	AchR/VGCC	33/f	CD19	Mobile by 2 months	Motte et al., 2024 [6]
MG/LES	AchR/VGCC	45/f	CD19	No wheelchair by 40 d	Motte et al., 2024 [6]
MG	AchR	37/f	CD19	CR by 4 months	Haghikia et al., 2024 [7]
LES	VGCC	54/m	CD19	Significant recovery	Wickel et al., 2024 [8]
DM	nr	12/m	CD19	Significant recovery	Nicolai et al., 2024 [10]
DM	MDA5	nr/f	CD19	Significant recovery	París-Muñoz et al., 2025 [24]
IMNM	SRP	33/m	CD19	PR, SRP-9, SRP-72, SRP-54, and Ro-52 decreased	Volkov et al., 2024 [11]
IMNM	SRP	42/f	CD19	PR	Wang et al., 2024 [20]
ASS	Jo-1	49/m	CD19	Significant recovery	Pecher et al., 2023 [13]
ASS	Jo-1	54/f	CD19	Clinical and CK, CRP, ESR, myoglobin and ferritin normalized	Haase et al., 2025 [14]
ASS	Jo-1	43/f	CD19/BCMA	2 relapses under CTCT	Müller et al., 2025 [22]
ASS	Jo-1	44/f	CD19	Major improvement	Taubmann et al., 2024 [23]
IIM	nr	43/f	CD19	Improvement	Müller et al., 2024 [3]
CIDP	nr	30/m	BCMA	Relapse after 12months	Dong et al., 2025 [15]
CIDP	nr	65/m	BCMA	CR at 24months	Dong et al., 2025 [15]

**Table 2:** Clinical manifestations, comorbidities, laboratory findings, and adverse reactions to CTCT in the same patients as shown in Table 1 ANA: anti-nuclear antibodies; CRP: C-reactive protein; CRS: cytokine release syndrome; CSF: cerebrospinal fluid; ICANS: immune effector cell-associated neurotoxicity syndrome; IL: interleukin; nr: not reported; NMD: neuromuscular disorder; MG: myasthenia gravis; LES: Lambert-Eaton syndrome; GABA-B: gamma-aminobutyric acid-B; DM: dermatomyositis; IMNM: immune-mediated necrotizing myopathy; ASS: anti-synthetase syndrome; IIM: idiopathic immune myositis; CIDP: chronic inflammatory demyelinating polyneuropathy; CTCT: chimeric antigen receptor T cell therapy

NMD	Clinical manifestations	Comorbidities	Laboratory findings	Adverse effects	Reference
MG	Dyspnea, quadriparesis, head drop	Thymoma	AchR antibodies (7.4 mmol/L)	Conjunctivitis, respiratory infection	[4]
MG	Generalized weakness, respiratory insufficiency	AB thymoma	Increased IL-2R, IL-4, IL-5, IL-6, IL-10	Pneumonia	[5]
MG	Bulbar paralysis, limb weakness	nr	Increased CRP, IL-6	Neutropenia, lymphopenia	[5]
MG/LES	Generalized weakness, double vision, respiratory insufficiency	nr	Vital capacity 31%	CRS 2 (flu-like symptoms, arterial hypotension)	[6]
MG/LES	Double vision, generalized weakness, respiratory insufficiency	Autonomic dysfunction (dry mouth, constipation)	Reduced vital capacity	Cervical lymph node swelling	[6]
MG	Generalized weakness	Rheumatoid arthritis	ANA 1:1,320	CRS 1	[7]
LES	Quadriparesis, bulbar symptoms	Graves’ disease, immune encephalitis	GABA-B antibodies	CRS 2	[8]
DM	Proximal limb weakness	None	Creatine-kinase	CRS 1, anemia, neutropenia	[10]
DM	Muscle weakness	Interstitial lung disease	MDA5 antibodies	None	[24]
IMNM	Proximal muscle weakness	None	Creatine kinase 5,900 U/L	No CRS, no ICANS	[11]
IMNM	Cervical and proximal muscle weakness	nr	Creatine kinase 10,000 U/L	CRP	[20]
ASS	Muscle weakness, myalgia	Interstitial lung disease	IL-6, IL-2R	nr	[13]
ASS	Fever, fatigue, myalgia, muscle weakness	Interstitial lung disease	Creatine kinase 8,800 U/L	CRS 2	[14]
ASS	Myalgia, fever, arthritis	Interstitial lung disease	Creatine kinase 5,000 U/L	CRS 1	[22]
ASS	Myalgia	Polyarthritis	ANA 1:10,000	ICANS 1	[23]
IIM	Myalgia, arthralgia	Lung disease	Creatine kinase	CRS 1, ICANS 1	[3]
CIDP	Relapsing symmetrical diffuse limb weakness	Psoriasis	CSF protein	CRS 1	[15]
CIDP	Relapsing symmetrical diffuse limb weakness	None	CSF protein	CRS 1	[15]

In a study of two patients with highly relapsed and refractory MG, one of whom had positive antibodies to the acetylcholine receptor (AchR) and the other antibodies to muscle-specific tyrosine kinase (MUSK), CAR T cells targeting the B cell maturation antigen (BCMA) were used and resulted in sustained clinical improvement over 18 months [5]. Therapeutic efficacy is thought to be based on the reconstitution of B cell lineages with persistently reduced pathogenic autoantibodies [5]. To determine the pathophysiology underlying the therapeutic efficacy of CAR T cells in these patients, longitudinal single-cell RNA and TCR sequencing was performed on serial post-infusion blood samples and on the corresponding infusion products [5]. Proliferating cytotoxic CD8 clones were found to be the main effectors of autoimmunity, while an impaired cytotoxic and proliferation signature and profound mitochondrial dysfunction in CD8+ T cells before infusion and subsequently defective CAR T cells after manufacturing could explain their characteristics in these patients [5].

In a study of two women with MG/LES overlap syndrome, CD19 CTCT was performed and resulted in rapid clinical recovery and regained mobility [6]. Profound depletion of B cells and normalization of AchR and voltage-gated calcium channel (VGCC) antibody levels were reflected in return to daily living and return from wheelchair dependence to cycling and hill walking [6]. These positive effects remained stable at follow-up after four and six months. The treatment was tolerated without major side effects [6].

In a 37-year-old woman with AchR antibody-positive MG and concurrent rheumatoid arthritis treated with pyridostigmine, glucocorticoids, and eculizumab, the Quantitative MG Score and MG Activity of Daily Living Score and Disease Activity Score remained active [7]. It was, therefore, decided to start CD19-directed CTCT [7]. Under this therapy, the patient regained her muscle strength and was able to jog for one hour, and all other symptoms disappeared [7]. Circulating B cells were undetectable on hospital day 4 and began to slowly reconstitute by day 150, and MG activity rapidly decreased, leading to complete remission of the disease [7]. The case showed that MG and rheumatoid arthritis respond to CTCT and that antibodies against citrullinated proteins can seroconvert [7].

Lambert-Eaton Syndrome

In addition to the two patients with MG and concomitant LES reported by Motte et al. and discussed in [6], a third patient with LES was reported who underwent CD19-targeted CTCT [8]. This patient was a 54-year-old man with idiopathic LES that manifested as progressive symmetric quadriparesis with severe gait disturbance and additional dysarthria, dysphagia, double vision, and tremor [8]. He had a high titer of VGCC type P/Q, but no malignancy was detected on repeated testing. Autologous anti-CD19 CTCT resulted in expansion of predominantly CD4+ CAR T cells with a terminally differentiated, CD45RA (TEMRA)-like effector memory cell phenotype suggestive of cytotoxic capabilities and subsequent B cell depletion [8]. VGCC antibody titers decreased, which was associated with an eightfold increase in walking distance [8]. The only relevant side effect was CRS and intermittent neutropenia [8].

Idiopathic Inflammatory Myopathies

IIM encompass a spectrum of autoinflammatory diseases characterized primarily by muscle inflammation and secondarily affecting various organs such as joints, skin, lungs, heart, and gastrointestinal tract [19]. Several myositis-specific and myositis-related autoantibodies have been identified, and based on clinical, serologic, and histopathologic features, IIM can be classified into several subgroups such as DM, anti-synthetase antibody (ASA), IMNM, inclusion body myositis, polymyositis, and overlap myositis [19]. The treatment of these diseases represents a major challenge and is often associated with great suffering for those affected. CTCT has been used in patients with DM [10], IMNM [11], and ASA [12-14].

Dermatomyositis

CTCT targeting B cells is not only beneficial in adult autoimmune diseases but has also been shown to be useful in juvenile autoimmune diseases [9]. However, there are specific challenges to consider in juvenile autoimmune diseases, such as long-term side effects, increased disease activity, and the need to reduce glucocorticoid exposure [9]. The first pediatric patient to undergo CTCT for the treatment of immune neuromyopathy was a 12-year-old male with severe, chronically active juvenile DM who failed to respond to multiple immunosuppressive lines of treatment, including B cell depletion with rituximab [10]. Following a single infusion of a fresh, autologous second-generation anti-CD19 CAR T cell product, complete B cell depletion was documented in the blood on day 5 after CTCT and in the bone marrow at week 2 [10]. The patient experienced minor side effects such as fever in the setting of mild CRS, transient anemia, and neutropenia, but no infections or neurotoxicity were observed [1]. Subsequently, the patient showed remarkable progressive improvement that persisted even after B cell recovery, as shown by the Childhood Myositis Assessment Scale, Cutaneous Assessment Tool for Myositis, laboratory tests, and MRI [10]. The patient achieved sustained B cell depletion and persistent clinical and radiologic improvement without immunosuppressants eight months after CTCT [10]. There is also a female child with MDA5-positive DM that benefited from CTCT [24].

Immune-Mediated Necrotizing Myopathy

The first patient with IMNM to receive CTCT was a 33-year-old male who presented with a 23-month history of IMNM diagnosed due to proximal muscle weakness, serum creatine kinase elevated to a maximum level of 5,900 U/L, and anti-SRP antibody positivity [11]. Initially, the patient received glucocorticoids as well as monthly IVIG and weekly methotrexate [11]. Due to persistent symptoms, the patient was switched to rituximab, with the last dose administered approximately eight months prior to CTCT. Due to persistent symptoms on rituximab, the patient received 4-1BBz anti-CD19 CTCT (CABA-201) [11]. The infusion product consisted predominantly of CD4+ effector memory T cells and exhibited cytolytic activity in vitro [11]. CABA-201 expansion peaked on day 15, preceded by an IFN-γ peak in serum on day 8 and peak serum IL-12p40 and IP-10 levels on day 15 [11]. No CRS or ICANS developed. Peripheral B cells were rapidly depleted after infusion. Peripheral B cells returned two months after infusion and were almost completely in transition. After infusion, muscle strength improved, creatine kinase levels decreased, and autoantibodies to SRP-9, SRP-72, SRP-54, and Ro-52 decreased compared to baseline, while antibodies associated with pathogens and vaccinations remained stable [11].

The second patient was a 42-year-old woman with treatment-resistant SRP-IMNM who received CD-19-targeted CTCT [20]. The patient showed significant clinical improvement one month after CTCT, as assessed by the Total Improvement Score, and experienced complete remission after two months, which persisted for six months [20]. Creatine kinase decreased from 2,295 U/L at the time of treatment to 1,383 U/L at the one-month follow-up [20]. There was also a significant improvement in the overall assessment by the doctor and the overall assessment by the patient from day 14 after treatment [20].

Anti-synthetase Syndrome

ASS is a rare autoimmune disease that causes inflammation in various body regions, including muscles, lungs, and joints [21]. ASS is characterized by specific autoantibodies, so-called ASA, which are often directed against the Jo-1 protein. The most common symptoms include muscle weakness, interstitial lung disease (ILD), and arthritis. Raynaud's syndrome and skin changes are also common [21]. The combination of ASS with myositis, arthritis, and ILD is present in up to 20% of patients at the onset of the disease. Studies have shown that patients with anti-Jo-1 autoantibodies can develop ILD in up to 90% of cases [12]. To date, CTCT has been performed in four patients with ASA [12,13,22,23].

The first patient is a 49-year-old man with ASS who was treated with glucocorticoids, methotrexate, leflunomide, baricitinib, rituximab, azathioprine, and mycophenolate before deciding to undergo CTCT due to progressive myositis and refractoriness to previous therapies [13]. Following CTCT, rapid clinical improvement was observed, and eight months later, Physician Global Assessment scores, muscle and pulmonary function tests, and muscle MRI showed no evidence of myositis [13]. Muscle enzymes, CD8+ T cell subsets, and the secretion of inflammatory cytokines in peripheral blood mononuclear cells (interferon gamma, interleukin-1 (IL-1), IL-6, and IL-13) had normalized [13]. In addition, there was a reduction in anti-Jo-1 antibody levels and a partial recovery of IgA (to 67% of normal), IgG (to 87%), and IgM (to 58%) levels [13].

The second patient is a 54-year-old woman diagnosed with ASS at the age of 52. The diagnosis was based on the clinical picture (polyarthralgia, myalgia, fever, fatigue, and skin changes); positive results for ANA (1:320), Jo-1, PL-12, and Ro52; an MRI of the thigh muscles consistent with muscle edema; a histology of the vastus lateralis muscle showing strong perifascicular enhanced major histocompatibility complex (MHC) class I and II positivity; endomysial infiltration; some necrotic myofibers in the perifascicular area; and a CT scan of the chest showing dorsobasal interstitial reticular findings and ground-glass opacities, as well as traction bronchiectasis consistent with a non-specific pattern of interstitial pneumonia [14]. The patient was subsequently treated with methotrexate, which was replaced by mycophenolate mofetil shortly after ILD was diagnosed [14]. Despite this treatment, high disease activity persisted, including steroid dependence, for the following six months. She was subsequently treated with various T and B cell-targeting agents such as cyclophosphamide, rituximab, tacrolimus, and daratumumab, which led to only temporary improvement [14]. Due to recurrent relapses under this treatment, she underwent CTCT (KYV-101) at the age of 54 [1]. Throughout the clinical course after the infusion, the patient showed continuous improvement in muscle strength, arthritis, and overall disease activity [14]. During the first month after CTCT, oral prednisolone treatment was reduced to 5 mg per day. MRI showed regression of the edematous changes, pulmonary function tests also improved, and pulmonary diffusion capacity showed a steady increase from 45% to 69% [14]. CTCT was well tolerated, with only mild CRS and no ICANS [14].

The third patient, a 45-year-old woman with refractory Jo1-associated ASS, received CD19-targeted CTCT [22]. She initially achieved remission but relapsed after nine months [22]. After reinfusion of the same product, the CAR T cells remained ineffective, and T cells directed against CD19-CAR were detected [22]. After bridging with daratumumab, BCMA-targeted therapy was performed [22]. BCMA-CAR T cells expanded, eliminated plasma cells in lymphoid tissue, reduced autoantibody levels, and induced a stable drug-free remission [22].

The fourth patient is a 44-year-old woman who tolerated CD19-targeted CTCT without complications and whose condition gradually improved over time [23]. Creatinine kinase decreased from 4,298 U/L at baseline to 99 U/L on day 150, myoglobin levels decreased from 2,945 to 53 mg/L, and alanine aminotransferase decreased from 317 to 37 U/L [23]. MRI of the thighs showed complete resolution of myositis, and the patient showed remarkable improvement in her physical function across all core measures of the International Myositis Assessment and Clinical Studies Group. She regained her muscle strength, achieving a manual muscle test score of 103/150 at baseline and full strength (150/150) at the final follow-up [23]. There was also one patient with IIM who underwent CD19-directed CTCT, but few clinical and biochemical details have been reported [3].

Chronic Inflammatory Demyelinating Polyneuropathy

To date, CTCT has been performed in two patients with CIDP [15]. Patient 1 was a 30-year-old man with motor-dominant CIDP and concomitant psoriasis [15]. Because the patient did not respond to steroids, IVIG, mycophenolate mofetil, azathioprine, and secukinumab, CD19-directed CTCT was performed. Before CTCT, he required assistance with eating and walking. After CTCT, the patient developed CRS, which was treated with steroids. At the six-month follow-up, he showed near-normal neurological function and regained independence in daily activities without assistance. However, 12 months after the infusion, he relapsed as a result of a severe COVID-19 infection (Table 1) [15]. Patient 2 was a 65-year-old man with CIDP according to the European Academy of Neurology (EAN) criteria who achieved sustained symptom remission for 24 months after CTCT. The patient maintained independence in daily activities and experienced only intermittent sensory deficits in the right toes. All neuropathy parameters showed improvement and remained stable over two years [15].

## Conclusions

CD19- or BCMA-targeted CTCT shows promising results in refractory MG, LES, IIM, and CIDP, offering sustained clinical improvement and a favorable safety profile. Previous case reports underscore the potential of CTCT therapy, especially in patients with inadequate response to B cell/plasma cell-depleting therapies. However, larger studies are needed to investigate the long-term efficacy, safety, and underlying mechanisms of CTCT in refractory immune-mediated NMDs. If the positive effect of CTCT is confirmed by such studies, CTCT could revolutionize the treatment landscape for immune NMDs.
